# FER1L4/miR‐372/E2F1 works as a ceRNA system to regulate the proliferation and cell cycle of glioma cells

**DOI:** 10.1111/jcmm.14198

**Published:** 2019-03-19

**Authors:** Liang Xia, Dekang Nie, Guangtao Wang, Caixing Sun, Gao Chen

**Affiliations:** ^1^ Department of Neurosurgery The second Affiliated Hospital of Medical College of Zhejiang University Hangzhou Zhejiang Province China; ^2^ Department of Neurosurgery Zhejiang Cancer Hospital Hangzhou Zhejiang Province China; ^3^ Department of Neurosurgery Yancheng First Peoples' Hospital Yancheng Jiangsu Province PR China

**Keywords:** ceRNA, E2F1, FER1L4, glioma, proliferation

## Abstract

Long non‐coding RNAs have recently become a key regulatory factor for cancers, whereas FER1L4, a newly discovered long non‐coding RNA, has been mostly studied in gastric carcinoma and colon cancer cases. The functions and molecular mechanism of FER1L4 have been rarely reported in glioma malignant phenotypes. In this study, it was found that the expression of LncRNA FER1L4 is upregulated in high‐grade gliomas than in low‐grade cases and that a high expression of LncRNA FER1L4 predicts poor prognosis of gliomas. Meanwhile, in vitro study suggests that expression of FER1L4 with SiRNA knockdown obviously suppresses cell cycle and proliferation. It is further demonstrated by experiments that the FER1L4 knockdown suppresses growth of in vivo glioma. Besides, it is found in our study that LncRNA FER1L4 expression is positively correlated with E2F1 mRNA expression. After knockdown of FER1L4 expression, E2F1 expression is significantly down‐regulated, whereas the expression of miR‐372 is significantly up‐regulated; the up‐regulation of miR‐372 leads to significant down‐regulation of FER1L4 and E2F1 expression. In addition, it is also found that FER1L4 can be used as competitive endogenous RNA to interact or bind with miR‐371 and thereby up‐regulate E2F1, thus promoting the cycle and proliferation of glioma cells. It may be one of the molecular mechanisms in which FER1L4 plays its oncogene‐like role in gliomas.

## 1 INTRODUCTION

Glioma is one of the commonest primary malignant nervous system tumours.^1^ Currently, although active surgery and adjuvant therapy have been used to treat it, the prognosis of most patients is still very poor.^2^ Particularly, in glioblastoma, the average survival time is still not more than 2 years.^1^ Studies have confirmed that the occurrence and development of glioma are closely related to the abnormal expression of various oncogenes or tumour suppressor genes.^3^ For example, recent studies have been focused on the clear correlation between IDH mutations,^4^ 1p19q combined deletion,^5^ MGMT methylation,^6^ TERT mutation,^7^ prognosis of glioma patients, glioma molecular typing or sensitivity to radiochemotherapy, which has been applied clinically.

Long non‐coding RNA (LncRNA) is a type of RNA that has a transcript longer than 200 nucleotides and that does not encode proteins.^8^ Studies have shown that LncRNA can regulate gene expression in epigenetics, transcriptional grade and post‐transcriptional grade and the regulating ways include chromosome modification, transcriptional activation and interference, etc.^9^ There is a close correlation between LncRNA and tumourigenesis as well as tumour development.^10^ Some LncRNAs show significant differences in expression between normal tissues and tumour tissues and abnormal LncRNA may play an important role in tumourigenesis.^11^ There is evidence to predict that LncRNA may become a new marker of tumour diagnosis and judgement, prognosis and therapeutic efficacy as well as a new target for tumour gene therapy.^12^

FER1L4, one of the important members of LncRNA, is closely related to tumourigenesis and tumour development.^13^ In recent years, several studies have confirmed that FER1L4 expression is closely related to colon cancer and gastric cancer.^14^, ^15^ The previous experimental study of our research group has preliminarily verified that FER1L4 is significantly higher in glioma cells than in astroglial cell lines and that FER1L4 is also closely related to proliferation, invasion and apoptosis of glioma cell lines.^16^ However, its expression in patients with glioma at different grades, its correlation with important clinical pathological factors and prognosis of patients with gliomas and the specific molecular mechanisms that LncRNA FER1L4 plays biological functions in glioma cells need further experiments to confirm currently.

In this study, it was found that the expression of LncRNA FER1L4 was significantly up‐regulated in high‐grade glioma patients compared with low‐grade expression. The knockdown of FER1L4 expression could significantly inhibit glioma cell proliferation and cell cycle. In vivo experiments confirmed that the down‐regulation of FER1L4 inhibited tumour growth in vivo. In addition, it is also found that FER1L4 can be used as competitive endogenous RNA (ceRNA) to adsorb E2F1 and thereby up‐regulate E2F1, thus promoting the cycle and proliferation of glioma cells. It may be one of the molecular mechanisms that FER1L4 plays its oncogene‐like role in gliomas.

## 2 MATERIALS AND METHODS

### 2.1 Human tissue specimens

All 47 glioma patients were aged between 23 and 75 and all were patients undergoing operation for the first time at the Department of Neurosurgery in Zhejiang Provincial Cancer Hospital during 2010‐2015. All patients underwent microsurgical total tumour resection and histopathology examination was performed. Two experienced pathologists diagnosed them as gliomas including 16 grade II, 14 grade III tumours and 14 grade IV tumours. All tumour tissues were surgically excised and placed in −80°C liquid nitrogen tubes for subsequent use. All patients underwent standardized radiotherapy and TMZ chemotherapy after neurosurgery. No radiotherapy and chemotherapy were performed before surgery. No patients received adjuvant immunotherapy. All patients provided written informed consent for the use of their tissues. This study was approved by the Ethics Committee of Zhejiang cancer hospital.

### 2.2 Cell culture

The gliomas S373‐MG, U251, U87‐MG, SHG‐44 and normal astrocytes 1800 derived from the Cell Library of the Chinese Academy of Sciences (PRC) were all respectively cultured in medium containing 10% fetal bovine serum, DMEM (Gibco BRL, USA) medium containing 1% penicillin (Gibco) and medium containing 5% CO_2_ at 37°C. Passaging and related operations were conducted after the degree of cell fusion reached 80%.

### 2.3 Cell transfection

The primers of FER1L4 were synthesized by Shanghai Invitrogen Biotechnology Co., Ltd. siRNA sequence was provided by Life Biotechnology Co., Ltd. The sense primer of siRNA sequence was 5‐CAGGACAGCUUCGAGUUAATT‐3 and the antisense primer was 5‐UUAACUCGAAGCUGUCCUGTT‐3. The sense primer of the control group was 5‐UUCUCCGAACGUGUCACGUTT‐3 and the antisense primer was 5‐ACGUGACACGUUCGGAGAATT‐3. One day prior to transfection, the cells were seeded in six‐well plates, with 4 × 10^5^ cells per well. 2 mL of medium containing 10% serum was added and placed in an incubator to culture overnight and transfection wasperformed until the cell confluence reached 70%‐80%. 4 μg of plasmid and 10 μL of transfection reagent were added to each well and the empty vectors were transfected as the negative control and the medium was changed to serum‐free medium. The medium was changed 6 hours after transfection.

### 2.4 Real‐time quantitative PCR

Total RNA was extracted using Trizol and it was quantitated using UV spectrophotometer. RNA was reversely transcribed into cDNA and amplified using RT‐PCR according to the instructions given inthe reverse transcription kit (Fermentas, USA). FER1L4 primer sense strand: 5'‐CCGTGTTGAGGTGCTGTTC‐3; antisense strand: 5‐CCCATCCCAGGAGGTCACCT‐3. E2F1 primer sense strand: 5‐AGCGGCGCATCTATGACATC‐3; antisense strand: 5‐GTCAACCCCTCAAGCCGTC‐3. Internal reference GAPDH sense strand: 5‐AAGGTGAAGGTCGGAGTCAA‐3, antisense strand: 5‐AATGAAGGGGTCATTGATGG‐3. The PCR reaction conditions were: pre‐denaturation at 95°C for 2 minutes, 95°C for 30 seconds, 57.4°C for 30 seconds, 72°C for 30 seconds, 35 cycles and extension at 72°C for 10 minutes . The PCR product was electrophoresed with agarose gel and photographed using a luminescence imaging system. The ratio of the greyscale values of FER1L/GAPDH or E2F1/GAPDH was taken as the relative expression level of FER1L or E2F1.

### 2.5 CCK‐8 cell proliferation assay

Cells in the logarithmic growth phase were seeded in 96‐well plates, with 5000 cells per well and three replicates were set in each group. Twenty four, 48, 72 and 96 hours after transfection, 10 μL of CCK‐8 (10 μL, C0038; Beyotime Institute) solution was added to each well and incubated in the incubator for 2 hours. The absorbance of each well was determined with the enzyme‐linked immunosorbent detector at 450 nm and the growth of the cells was compared.

### 2.6 Cell cycle analysis

The cells in logarithmic growth phase were seeded and cultured in six‐well plates. Drug solution was added after the cells grew to 70%. After culturing for a period of time, they were digested with appropriate amounts of trypsin to prepare a single cell suspension, centrifuged at 150 *g*, washed twice with PBS and fixed with 75% cold ethanol at −20°C. Before machine determination, they were centrifuged to remove the ethanol, washed twice with PBS, filtered through 300 mesh nylon mesh and prepared to be cell suspension with 200 μL PBS suspension cells. 300 μL of PI dye solution was added and they were dyed in a dark environment at 4°C for more than 30 minutes; 10 000 cells were counted using the FACSCalibur, BD, USA and the proportion of each phase (G0/G1, G2/M, S) was finally statistically analysed.

### 2.7 Western blot analysis

The cells in logarithmic growth phase were lysed and the supernatant and the protein were extracted. After adjusting the protein concentration of each cell sample, SDS‐PAGE was performed on 20 L of each sample and primary antibodies against E2F1 (1:500; Cell Signaling, Danvers, MA, USA), P21 (1:1000; Sigma, USA), cyclin D2 (1:1000; Abcam, USA), P‐ERK (1:1000; Abcam) or β‐actin (1:2000; Beyotime Institute of Biotechnology, PRC) were used for incubation overnight under 4°C. Subsequently, horseradish peroxidase‐conjugated secondary antibody (1:500; Beyotime Institute of Biotechnology) was used for incubation. Then they were developed, washed and dried. After repeating Western blot several times and scanning the images with a gel image analyser, the ratio of the expression level of specific protein (strand gray scale) and that of internal reference (β‐actin) in each group was determined with the four‐star image processing system to take it as a parameter for their expression levels.

### 2.8 Xenograft model

Experimental animals: BALB/c mice (Shanghai Shrike Experimental Animal Company), 4‐6 weeks old. Eight nude mice were randomly divided into two groups, the Si‐FER1L4 group and control group respectively; cells in logarithmic growth phase were digested with trypsin and prepared as a single cell suspension, which was washed with serum‐free DMEM medium for three times. Each group of cells was injected into the left back of corresponding nude mice in each group and the number of cells injected was 1 × 10^5^/each mouse, 200 μL of serum‐free medium. The size of the tumour was measured with a vernier caliper and recorded every 7 days based on the formula: length × width × height × 0.5236. All the tumour‐bearing nude mice were killed 42 days after inoculation with the tumour cells. The subcutaneous tumours were taken to observe the sizes. The tumours were detached to measure their sizes, weigh them and record and photograph.

### 2.9 Luciferase assays

Glioma cells (4 × 10^4^) were seeded in triplicate in 96‐well plates and allowed to settle for 24 hours. MiR‐327 mimics, pGL3‐FER1L4‐wild and pGL3‐FER1L4‐mut vectors were purchased from RiboBio (RiboBio Co. Ltd, Guangzhou, Guangdong) and were respectively co‐transfected with U251 and U373MG cells as experimental groups and the group with only transfected vector cells was taken as the control group. The transfection efficiency was tested to determine successful transfection. The luciferase assay was performed by using the dual‐luciferase reporter assay system (Promega) 48 hours after transfection. Three independent experiments were performed and the data are presented as the mean ± SD.

### 2.10 Statistical analysis

The relationship between the gene expression and clinical characteristics of patients was analysed using chi‐squared test and rank correlation analysis and the prognosis was analysed using K‐M survival analysis. The in vitro cell experiments were repeated for three times and the data were expressed as the mean ± SD. The *t* test was performed to compare the mean of the two groups. The two‐sample *t* test was applied to compare the means of the two groups. The ANOVA test was used to compare the means of the three groups. *P* < 0.05 was considered statistically significant. Statistical analysis was conducted using SPSS17.0 and graphpad prism 5 statistical software.

## 3 RESULTS

### 3.1 LncRNAs FER1L4 and E2F1 are highly expressed in high‐grade gliomas and their high expression predicts a worse prognosis of gliomas

**Figure 1:**
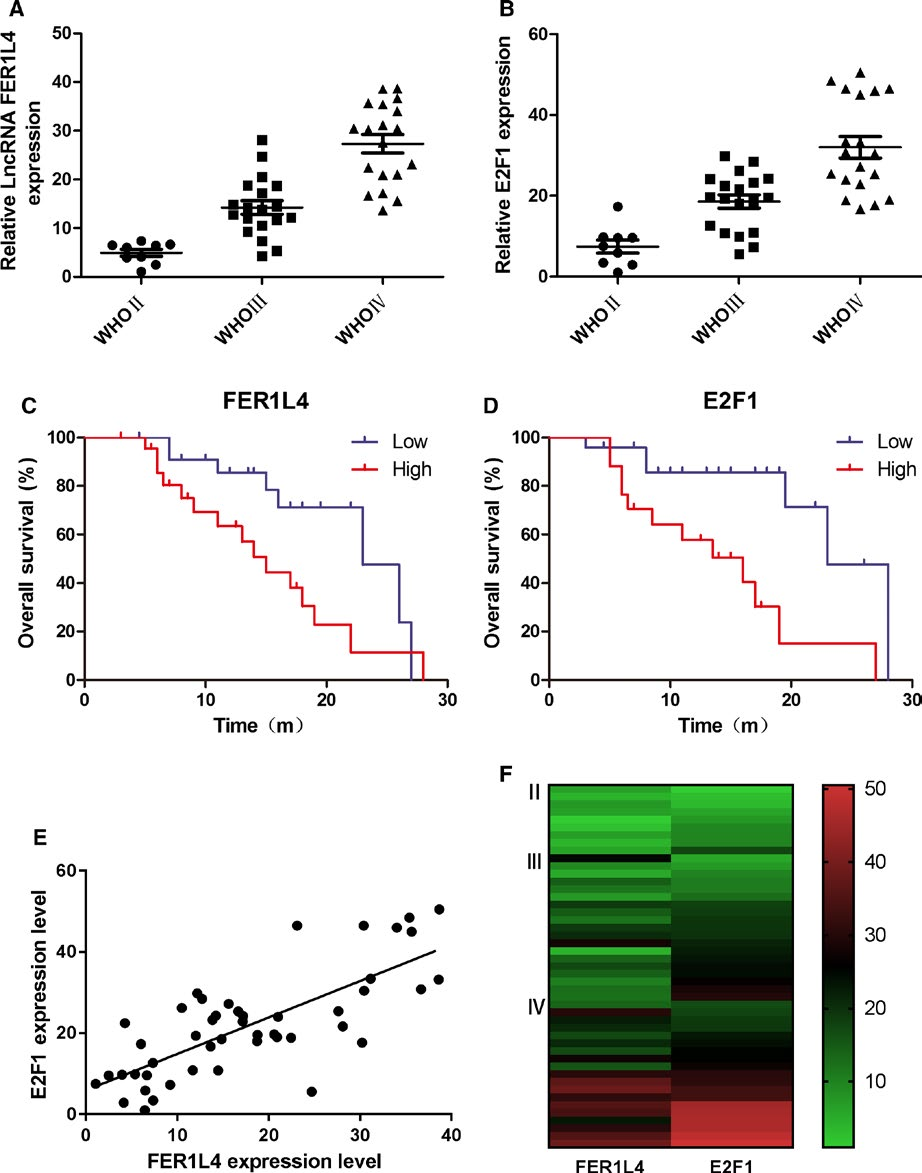
The LncRNAs FER1L4 and E2F1 expression in different grades of gliomas (WHO II‐IV). A, Expression of FER1L4 level in different grades of gliomas (WHO II gliomas, WHO III gliomas and WHO IV gliomas). B, Expression of E2F1 level in different grades of gliomas (WHO II gliomas, WHO III gliomas and WHO IV gliomas) (*P < 0.05, **P < 0.01, ***P < 0.001). C and D, Kaplan‐Meier post‐operative survival curve for patterns of patients with glioma and FER1L4 and E2F1 expression. E, The linear correlations between the expression level of FER1L4 and E2F1 in different grades of gliomas (*R* = 0.573, ***P < 0.001). The data were obtained using the logistic regression analysis. F, The heat map between the expression level of FER1L4 and E2F1 in different grades of gliomas.

The expression of LncRNAs FER1L4 and E2F1 in gliomas at 47 different grades was detected using RT‐PCR and the results showed that the expression of FER1L4 in different grade WHO IV glioma tissues (27.32 ± 1.90) was dramatically higher than that in grade WHO III gliomas (14.24 ± 1.41) (*P* < 0.001). A significant difference was also found between WHO III gliomas (14.24 ± 1.41) and grade WHO II (4.95 ± 0.72) (*P* < 0.01) (Figure 1A). Similarly, E2F1 was significantly higher in grade WHO IV gliomas (32.01 ± 2.67) than in grade WHO III gliomas (18.57 ± 1.64) and grade WHO II (7.443 ± 1.64) (*P* < 0.001) and with the increase in the grade, its expression also showed a gradually increasing trend (Figure 1B).

**Figure 2:**
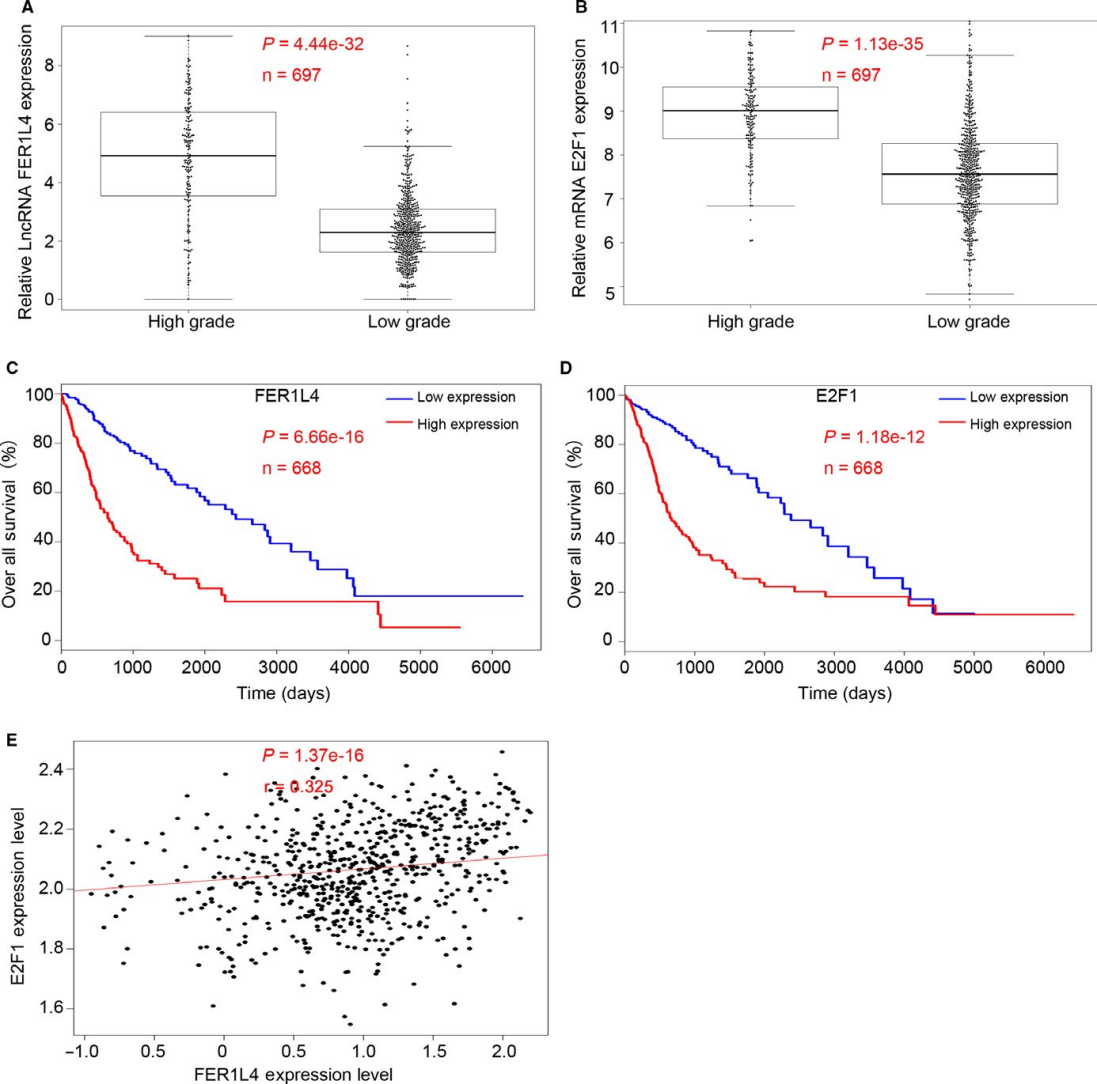
The LncRNAs FER1L4 and E2F1 expression in different grades of gliomas (WHO I‐IV) from TCGA database. A and B, Expression of FER1L4 (A) and E2F1 (B) level in different grades of gliomas (low grade: WHO I‐ II gliomas; high grade: WHO III‐IV gliomas) (***P < 0.001). C and D, Kaplan‐Meier post‐operative survival curve for patterns of patients with glioma and FER1L4 and E2F1 expression (***P < 0.001). E, The linear correlations between the expression level of FER1L4 and E2F1 in glioma tissues from TCGA database (*R* = 0.325, *P* < 0.001). The data were obtained using the logistic regression analysis.

In addition, we divided the expression of FER1L4 or E2F1 into high expression group and low expression group according to the median of FER1L4 or E2F1 expression. The results suggested a significant correlation between the expression of FER1L4 and E2F1 and the prognosis of glioma patients and the high expression of FER1L4 (*P* < 0.05) or E2F1 (*P* < 0.01) predicts a worse prognosis of gliomas (Figure 1C and D). Further correlation analysis indicated that the expression of FER1L4 and E2F1 showed a significant positive correlation (*P* < 0.001) (Figure 1E and F). Bioinformatics further proved the results of our experimental studies (Figure 2).

### 3.2 The knockdown of FER1L4 with SiRNA was showed to inhibit the proliferation and cell progression of glioma cells

**Figure 3:**
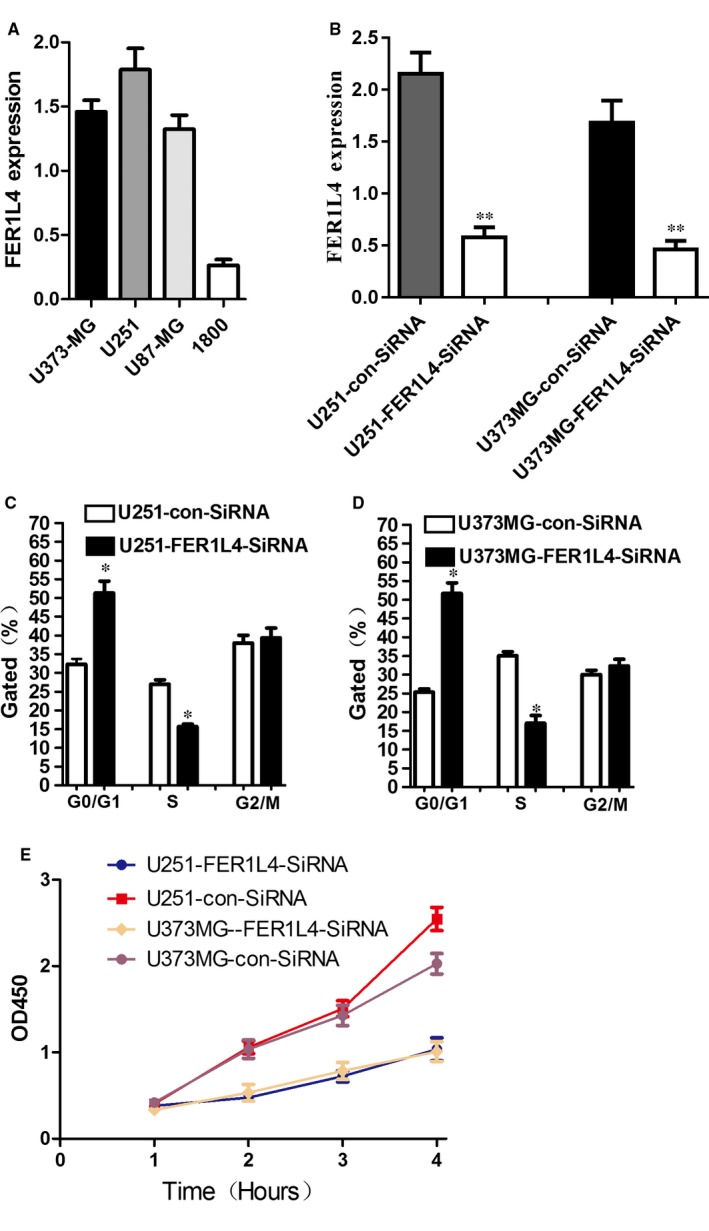
Effects of FER1L4 knockdown on the proliferation and cell cycle of glioma cells. A, Expression of FER1L4 level in three glioma cell lines (U373MG, U251, U87) and one normal astroglia cell line (1800) (*P < 0.05). (B) Extent of knockdown of FER1L4 transcription upon transfection of siRNA sequence in comparison with the negative control (con‐siRNA). C and D, Flow cytometry quantitation of cell cycle progression in glioma cells (U251 and U373MG). E, Growth curves indicating cell growth promotion by CELF1 (U251 and U373MG).

In order to further investigate the role of FER1L4 in glioma cells, RT‐PCR was used to determine the expression of FER1L4 in glioma cells U87, U251, U373MG and normal astrocyte 1800. The results showed that the expression of FER1L4 in three cell lines of gliomas was significantly higher than that of normal astrocyte 1800 (*P* < 0.05) and that of FER1L4 was more highly expressed in U251 and U373MG cells (Figure 3A). SiRNA‐FER1L4 was transfected into glioma U251 and U373MG cells and the results indicated that it could significantly knock down the expression of FER1L4 (*P* < 0.01) (Figure 3B). In addition, CCK‐8 and flow cytometry was used to determine the changes in the cell proliferation and cycle after the knockdown of FER1L4 expression. The results suggested that the SiRNA knockdown on FER1L4 can inhibit the proliferation and cycle of glioma cells (*P* < 0.05) (Figure 3C‐E).

### 3.3 In vivo experiments confirmed that the knockdown of FER1L4 expression can inhibit the growth of subcutaneous tumours in nude mice

**Figure 4:**
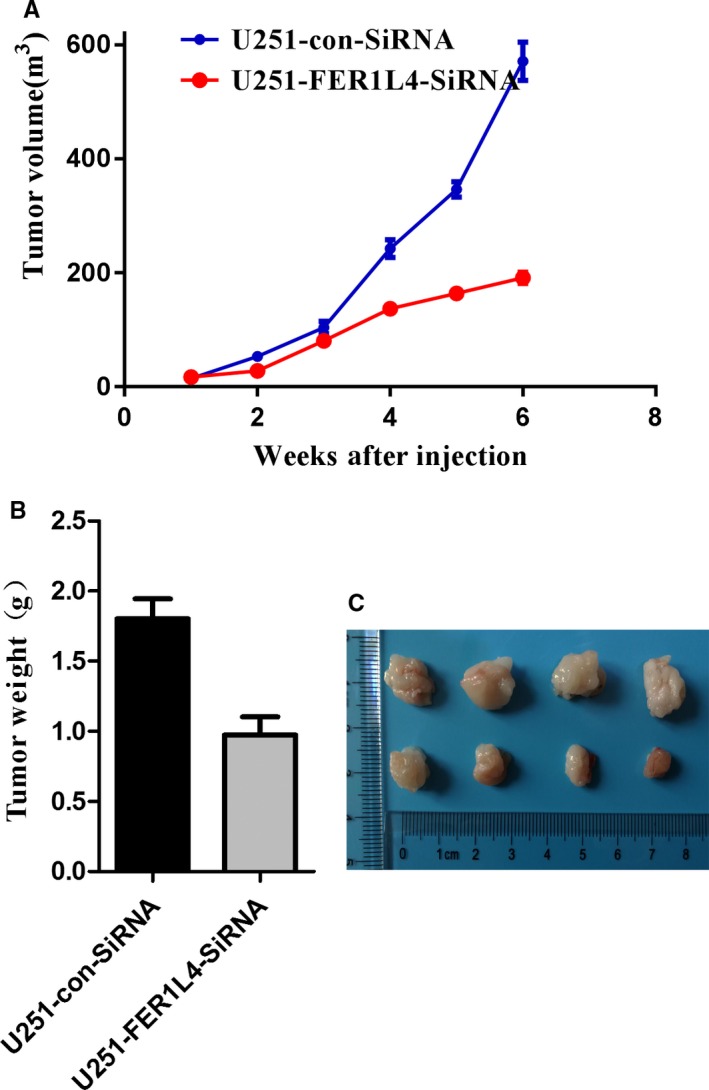
FER1L4 promotes the growth of xenograft glioma tumour in vivo. A, The curve shows the tumour growth of cells with FER1L4 knockdown, negative control (con‐siRNA). B and C, The tumour tissues were harvested and individually weighed at 6 wk when mice were killed (*P < 0.05)

In order to further explore the effect of FER1L4 expression on the proliferation of gliomas in vivo, glioma U373MG cells were transfected by stabilizing FER1L4 siRNA. Compared with the non‐transfected control group, after 14 days of G418 deletion, some of the SiRNA‐FER1L4 cells survived. WB experiment verified that FER1L4 was down‐regulated by 64% in the stably transfected cells compared with the control cells (1 × 10^6^ per mouse) which were subcutaneously injected into the tibia of nude mice to evaluate the effect of FER1L4 on glioma production in vivo. Tumour volume was evaluated each week and the measurement data was recorded. The data showed a significant decrease in the tumour volume in the SiRNA‐FER1L4 group as compared with the control group (Figure 4A). Until the sixth week, the nude mice were killed and the subcutaneous tumours were removed. Then the tumours were weighed. The results showed that the weight of tumours in the SiRNA‐FER1L4 group was significantly lower than that in the control group (*P* < 0.05) (Figure 4B and C).

### 3.4 The change in FER1L4 expression has effect on the expression of E2F1, downstream genes ERK, P21 and cyclinD2

**Figure 5:**
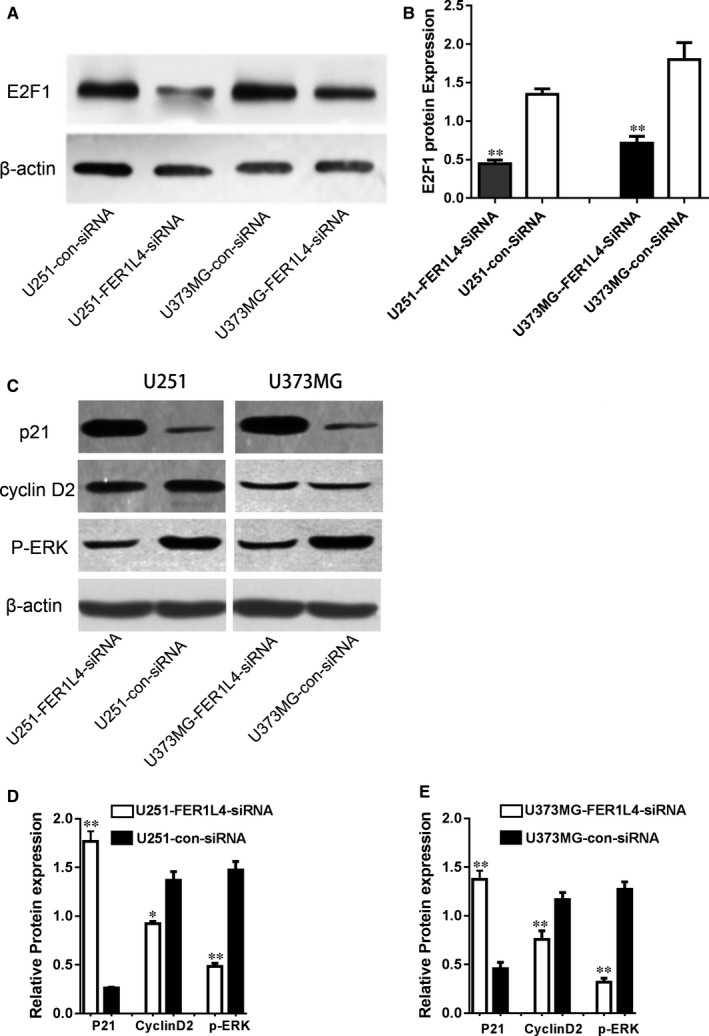
The lncRNA FER1L4 regulates target gene expression through E2F1. A and B, Western blot of the RNA level of E2F1 in glioma cells (U251 and U373MG) transfected with FER1L4‐siRNA or control siRNA (**P < 0.01). C‐E, Western blot analysis of the expression level of target genes (p21, cyclin D2, P‐ERK) in glioma cells (U251 and U373MG) transfected with FER1L4‐siRNA or control siRNA (*P < 0.05, **P < 0.01)

The study above showed that the expression of FER1L4 and E2F1 in different grades of glioma tissues showed a significant positive correlation. The results suggest that there may be some regulatory relationship between FER1L4 and E2F1. Therefore, E2F1 expression was determined by using Western blot when the expression of FER1L4 was down‐regulated by SiRNA, which showed that the protein expression of E2F1 was significantly down‐regulated (Figure 5A and B). To further investigate the mechanism of FER1L4‐mediated E2F1 regulation on proliferation and cycle of glioma cells, the changes in the protein expression of E2F1 downstream molecules P21 and cyclinD2 and ERK phosphorylation by interfering with FER1L4 were examined. The results showed that after interfering with FER1L4, the E2F1 expression was down‐regulated, the P21expression was up‐regulated, the cyclinD2 expression was significantly down‐regulated and the ERK phosphorylation was significantly reduced (Figure 5C‐E) suggesting that P21 can be up‐regulated and that cyclinD2 and ERK phosphorylation can be down‐regulated by down‐regulating FER1L4 targeted E2F1 in glioma cells, so as to inhibit the growth and proliferation of glioma cells.

### 3.5 FER1L4 regulates glioma cell proliferation by ceRNA mechanism

**Figure 6:**
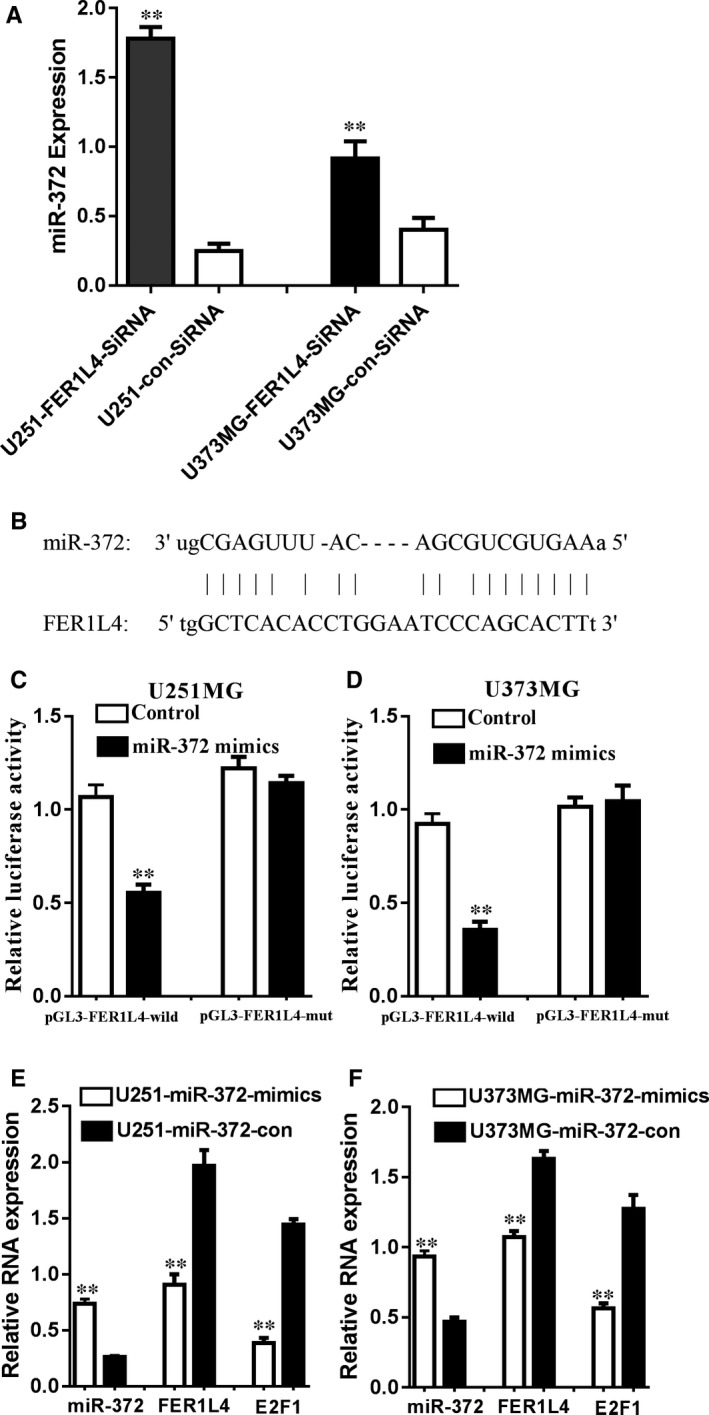
The lncRNA FER1L4 increases E2F1 expression by inhibiting miR‐372. A, Knockdown of FER1L4 increased miR‐372 expression both in glioma cells (U251 and U373MG) (**P < 0.01). B, Schematic diagram of the predicted binding sites between miR‐372 and FER1L4. C and D, Luciferase activity in U251 (C) and U373MG (D) cells co‐transfected with miR‐372 mimics and luciferase reporters containing control vector, pGL3‐FER1L4‐wild, and pGL3‐FER1L4‐mut (**P < 0.01). E and F, miR‐372 overexpression down‐regulated the endogenic FER1L4 and E2F1 expression in glioma cells (U251 and U373MG) (**P < 0.01)

The miRNAs that constitute ceRNA pattern with FER1L4 and E2F1 were searched from lnceDB database. The Reference Sequence of FER1L4 as well as E2F1 was obtained from GenBank and that of miRNA was obtained from miRbase. Possible bindings between FER1L4, E2F1 and miRNA were respectively predicted based on Miranda and the miRNAs likely to bind both FER1L4 and E2F1 included: hsa‐miR‐4262, hsa‐miR‐346, hsa‐miR‐93, hsa‐miR‐326, hsa‐miR‐181, hsa‐miR‐520,hsa‐miR‐367, hsa‐miR‐4644, hsa‐miR‐329, hsa‐miR‐874, hsa‐miR‐372, hsa‐miR‐485, hsa‐miR‐4306 and hsa‐miR‐302, with a total of 14 miRNAs. FER1L4 was interfered by siRNA and the PCR results showed that hsa‐miR‐372 expression was significantly up‐regulated (*P* < 0.01) (Figure 6A). The fluorescein‐labelled test further confirmed the direct interaction between FER1L4 and hsa‐miR‐372 (Figure 6B‐D). In addition, studies have shown that in breast cancer cells, hsa‐miR‐372 has been identified to inhibit tumour proliferation through direct regulation on E2F1.^17^ Therefore, it is speculated that FER1L4/hsa‐miR‐372/E2F1 may serve as a regulatory mechanism of ceRNA for glioma cell proliferation. Further over‐expression of hsa‐miR‐372 indicated the down‐regulation of both FER1L4 and E2F1 expression (Figure 6E and F). The results further proved that as a ceRNA, FER1L4/hsa‐miR‐372/E2F1 regulates the molecular mechanism of glioma cell proliferation.

## 4 DISCUSSIONS

Glioma is a highly malignant primary nervous system tumour.^2^ The prognosis of most patients is still very poor in spite of the active surgical treatment and adjuvant therapy.^18^ Particularly, the average survival time of glioblastoma is still not more than 2 years.^19^ Current studies show that the occurrence and development of gliomas are closely related to the abnormal expression of various oncogenes and tumour suppressor genes.^20^ However, the precise molecular mechanism for its development is not very clear. Therefore, it is of great significance to further explore the relationship between the changes in new gene function and the development and malignant characteristics of glioma tumours, to reveal the precise molecular mechanism of its occurrence and development, to develop reasonable therapeutic drugs and determine the prognosis and to further improve the treatment of glioma and the prognosis of glioma patients.^3^

The ceRNA hypothesis has been paid attention to by academic community after being proposed, as it represents a brand new mode of post‐transcriptional gene expression regulation.^21^ A number of studies have shown that lncRNAs can participate in the regulation of various biological processes through ceRNA mechanism, such as muscle differentiation, cell resistance, neural cell differentiation, etc.^22^ In addition, lncRNA, as ceRNA, plays an important role in carcinogenesis.^23^ For example, in ovarian cancer, lncRNA HOST2 up‐regulates the expression of HMGA2 and c‐MYC by the competitive binding with let‐7b‐5p, thereby promoting the occurrence and development of ovarian cancer.^24^

FER1L4, as a newly discovered LncRNA, is mainly focused on gastric cancer and colon cancer in current studies.^13^, ^14^ In the study of Liu et al, it was first confirmed that corresponding to paracarcinoma tissues, the expression of FER1L4 in 91.8% gastric cancer tissues was significantly down‐regulated and the low expression of FER1L4 was significantly correlated with the tumour volume, malignancy, lymphatic and distant metastasis, depth of invasion, TNM stage, vascular nerve infiltration and serum CA72‐4 expression of gastric cancer.^25^ Therefore, the results confirmed that the expression level of FER1L4 can be taken as an excellent and important indicator for the early diagnosis of gastric cancer. Subsequent study has shown that FER1L4 can be used as an important endogenously‐competent RNA and plays a role of tumour suppressor gene in colon cancer. Its expression is negatively correlated with the expression of miR‐106a‐5p, both of which are closely related to the depth of invasion, vascular invasion, lymph node metastasis and clinical stage of colon cancer.^14^ The exogenous increase in the expression of FER1L4 can significantly down‐regulate the expression of miR‐106a‐5p and influence the proliferation, migration and invasion of colon cancer.^14^ The above study has confirmed that FER1L4 may play a role of tumour suppressor gene in gastric cancer and colon cancer and its expression level can be taken as an excellent indicator for the early diagnosis and prognosis of gastric cancer and colon cancer.

The previous experimental study of this subject has preliminarily confirmed that FER1L4 is significantly higher in glioma cells than in astrocytes cell lines. FER1L4 can significantly promote the proliferation, invasion and apoptosis of glioma cell lines.^16^ Our study is inconsistent with the results of studies on gastric cancer and colon cancer indicating that FER1L4 may play an inconsistent role in different tumour cells.^16^ In this study, it is found that FER1L4 was highly expressed in high‐grade gliomas in different grades of glioma tissues and its high expression was significantly negatively correlated with the prognosis of glioma patients. The knockdown of FER1L4 expression significantly inhibited the proliferation of glioma cells, proving that FER1L4 plays an oncogene role in glioma cells.

The transcription factor E2F family consists of eight different family members, from E2F1 to E2F8.^26^ E2F1 was screened by Helin in 1992. It can be used as a transcription factor to regulate the cell cycle, but high expression of E2F1 can also induce many types of apoptosis.^27^ Therefore, E2F1 possesses double properties of carcinogenesis and apoptosis induction and its specific effects are related to the specificity of organ tissues and so on.^28^ In some tumours such as lung cancer and breast cancer, E2F1 promotes canceration.^29^, ^30^ Our study found that high expression of E2F1 appears in glioma tissues and it gradually increases with the rise in glioma WHO grade. Similarly, high expression of E2F1 predicts a poor prognosis of gliomas. There was a significant positive correlation between its expression in gliomas and FER1L4 expression suggesting a synergistic effect between FER1L4 and E2F1, which participate in the malignant biological process of glioma. Subsequently, by down‐regulating FER1L4, we confirmed that E2F1 expression was also down‐regulated and that FER1L4 and E2F1 were involved in the cell cycle of gliomas (FER1L4 regulates the cell progression in G2‐M by regulating E2F1).

Further experiments confirmed that FER1L4 can directly bind with miR‐372 and the down‐regulation on FER1L4 expression can significantly up‐regulate E2F1 expression and down‐regulate miR‐372 expression. Furthermore, in the previous literature in breast cancer cells, it has been clarified that hsa‐ miR‐372 can inhibit tumour proliferation through direct regulation on E2F1.^17^ Therefore, it is speculated that FER1L4/miR‐372/E2F1 may serve as a ceRNA regulatory mechanism for glioma cell proliferation. Further over‐expression of miR‐372 showed that the expression of FER1L4 and E2F1 was down‐regulated and the results further confirmed that as a ceRNA, FER1L4/miR‐372/E2F1 regulates the molecular mechanism of glioma cell proliferation.

## 5 CONCLUSIONS

In summary, there is evidence to speculate that Long Non‐Coding RNA FER1L4 may play an important role in the cycle and proliferation of gliomas cells and it may serve as a new indicator for prognosis prediction and malignancy judgement of gliomas. The results may improve our knowledge of the regulatory mechanism of LncRNA FER1L4 and provide useful indicators and methods for the clinical diagnosis and treatment of gliomas.

## 6 ETHICS APPROVAL AND CONSENT TO PARTICIPATE

Additional informed consent was obtained from all individual participants for whom identifying information is included in this article. Animal experiments were performed in strict accordance with the Institutional Animal Care guidelines of interest.

## CONFLICTS OF INTEREST

All the authors declare that they have no conflicts of interest.

## AUTHORS’ CONTRIBUTIONS

CG and SCX conceived the project and participated in the study design, supervision of laboratory processes analysis and interpretation of the results. XL conceived the writing of the manuscript. NDK and XL participated in the study design, helped in animal experiments and drafting the manuscript. NDK and XL helped in vitro experiments and data analysis. NDK participated in data interpretation and provided critical review in the manuscript preparation. All authors read and approved the final manuscript.
